# Tumour blood vessel normalisation by prolyl hydroxylase inhibitor repaired sensitivity to chemotherapy in a tumour mouse model

**DOI:** 10.1038/srep45621

**Published:** 2017-03-31

**Authors:** Satoshi Koyama, Shinji Matsunaga, Masaki Imanishi, Yoichi Maekawa, Hiroya Kitano, Hiromi Takeuchi, Shuhei Tomita

**Affiliations:** 1 Division of Molecular Pharmacology, Department of Pathophysiological and Therapeutic Science, Tottori University Faculty of Medicine, Japan; 2 Division of Otolaryngology, Head and Neck Surgery, Department of Sensory and Motor Organs, Tottori University Faculty of Medicine, Japan; 3 Department of Pharmacology, Osaka City University Graduate School of Medicine, Japan; 4 Department of Parasitology Gifu University Graduate School of Medicine, Japan; 5 Domain of Integrated Life Systems, Gifu Center for Highly Advanced Integration of Nanosciences and Life Sciences (G-CHAIN), Gifu University, Gifu, Japan

## Abstract

Blood vessels are important tissue structures that deliver oxygen and nutrition. In tumour tissue, abnormal blood vessels, which are hyperpermeable and immature, are often formed; these tissues also have irregular vascularisation and intravasation. This situation leads to hypoperfusion in tumour tissue along with low oxygen and nutrition depletion; this is also called the tumour microenvironment and is characterised by hypoxia, depleted nutrition, low pH and high interstitial pressure. This environment induces resistance to anticancer drugs, which causes an increase in anticancer drug doses, leading to increased side effects. We hypothesised that normalised tumour blood vessels would improve tumour tissue perfusion, resupply nutrition and re-oxygenate the tumour tissue. Chemotherapy would then be more effective and cause a decrease in anticancer drug doses. Here we report a neovascularisation-inducing drug that improved tumour vascular abnormalities, such as low blood flow, blood leakage and abnormal vessel structure. These results could lead to not only an increased chemo-sensitivity and tissue-drug distribution but also an up-regulated efficiency for cancer chemotherapy. This suggests that tumour blood vessel normalisation therapy accompanied by angiogenesis may be a novel strategy for cancer therapy.

Several studies have shown that tumour vessels are different from normal microvessels in both structure and function^1^. These tumour vessels have highly abnormal features, such as a serpentine course, irregular branching and arteriovenous shunt formation^2^. Blood flow through a tumour does not follow a constant, unidirectional path. Furthermore, tumour vessel endothelium has defects, such as poorly-connected tight junctions and sparse pericyte coverage^3^. Tight junctions manage the movement of solutes, ions and water across the paracellular space^4^. Therefore, a deficiency in tight junctions leads to vessel hyperpermeability, decreased nutrition and impaired oxygen delivery^5^. Due to a lack of tight junctions and pericytes, tumour tissue has low blood flow, poor perfusion and high blood leakage^6^. These features lead to challenging environment with hypoxia, acidosis, starvation and increased interstitial pressure; this is called the tumour microenvironment^7^. The tumour microenvironment is characterised by marked gradients in the cell proliferation rate and regions of hypoxia and acidity; these issues can substantially influence not only tumour response to anticancer drugs and radiation sensitivity but also drug distribution in tumour tissue^8^. The tumour microenvironment can induce both chemo- and radio-resistance^9^.

Hypoxia-inducible factors (HIFs) are key transcriptional factors that are cellular adaptive responses to hypoxia. They play a pivotal pro-survival role in ischaemic tissues. Therefore, there have been numerous reports of attempts to overcome ischaemic conditions by stabilising HIFs^10^,^11^,^12^. Local upregulation of HIF-1 promotes mobilisation and recruitment of endothelial progenitor cells (EPCs), thereby contributing to direct or indirect angiogenesis and vasculogenesis in ischaemic tissue^13^. HIFs are regulated by the prolyl hydroxylase domain protein (PHD); the activity of PHD depends on the oxygen concentration. Dimethyloxalyl glycine (DMOG), which is an inhibitor of PHD and a 2-oxoglutarate analogue, decreases the PHD hydroxylase activity and the stabilisation of HIFs. We had previously reported that DMOG administration improved ischaemia via angiogenesis and vasculogenesis through enhanced retention of EPCs in ischaemic random pattern skin flap model mice^13^. Therefore, we determined that the tumour microenvironment was similar to an ischaemic environment. Therefore, we hypothesised that stimulating the HIF signalling pathway following PHD inhibition may induce angiogenesis and vasculogenesis in tumour tissue and improve tissue blood flow, perfusion and the tumour microenvironment. These phenomena may enhance anticancer drug and radiation sensitivity in tumours and prove beneficial in cancer therapy.

The aim of this study was to assess whether PHD inhibitor administration induced normalisation of tumour vessel features, such as the vessel lumen, tight junction formations and pericyte coverage. We examined not only the functional features associated with the tumour microenvironment, such as tissue perfusion, vessel permeability and hypoxia, but also the features resulting in chemotherapy efficiency. Here we show that transient PHD inhibition led to the normalisation of tumour blood vessels, which could enhance anticancer drug sensitisation in a tumour mouse model.

## Results

### PHD inhibitor changed tumour vessel structures

**Figure 1:**
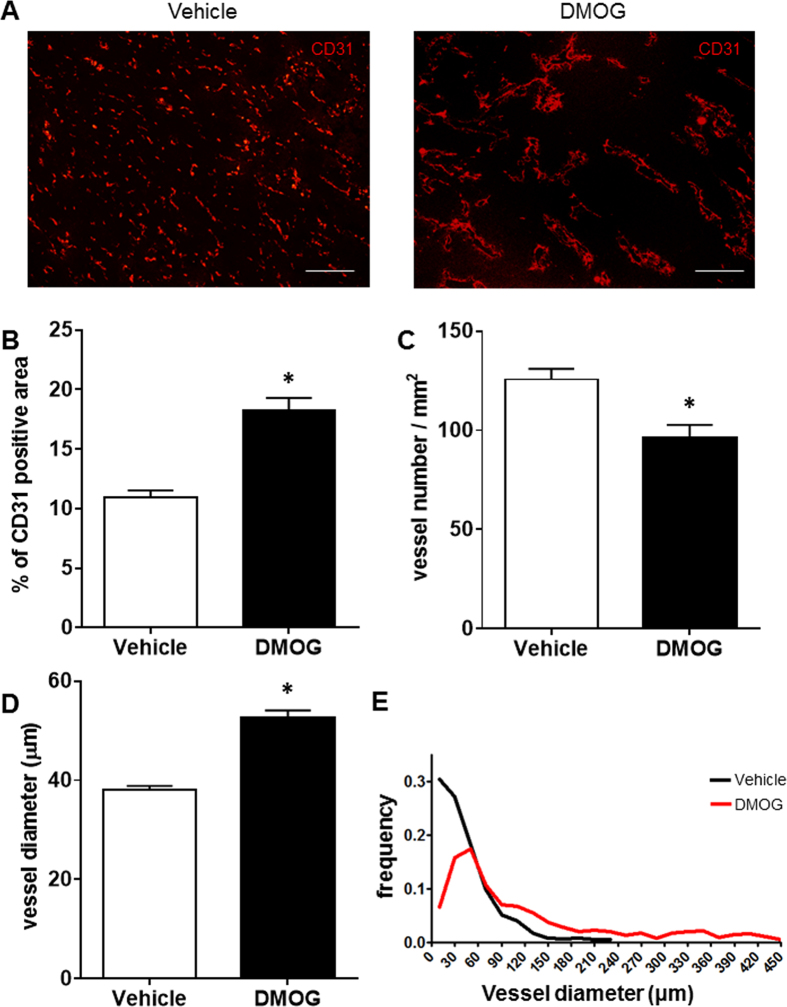
PHD inhibitors change tumour vessel aspect. The Lewis lung carcinoma cells were subcutaneously injected into the right flank of the mice. At 10 days after the tumour cell injection, the mice were administered dimethyloxalyl glycine (DMOG) intraperitoneally. At 7 days after the DMOG administration, the mice were sacrificed, and the tumours were excised. (**A**) Representative image of tumour endothelial cell staining. The endothelial cells were stained with CD31 antibody (red). (**B**) Quantification of CD31-positive area in the stained tumour tissues. These quantifications were performed using Image J software. (**C**) The vessel numbers were assessed from the CD31 tissue staining. (**D**,**E**) The vessel length was measured and calculated from the obtained images. The bar charts show means ± standard error of the mean (s.e.m). These pictures were taken over 15 parts in each tumour. n = 3 mice per group. *Shows *p*-values that were <0.001 in (**B)**, 0.3683 in (**C**) and <0.001 in D, respectively v.s. vehicle, Mann–Whitney U test. Scale bars represent 200 μm.

To study the effects of PHD inhibitor on tumour blood vessels, we administered DMOG to Lewis lung carcinoma (LLC) model mice. Microvessels consist of a single layer of endothelial cells and pericytes covered with an endothelial layer on the outside. However, tumour blood vessels usually lack structure. Therefore, we focused on the tumour blood vessels, particularly the endothelial cells (ECs), which were stained for CD31. The DMOG treatment drastically changed aspects of the tumour blood vessels; this slightly increased the CD31-positive area, markedly increased the average vessel diameter and decreased the blood vessel density (Fig. 1A–D). Moreover, the frequency of tumour vessel diameter was decreased in small diameter vessels and increased large diameter vessels (Fig. 1E). Furthermore, we assessed whether another PHD inhibitor FG4592 treatment, which is bound to 2-oxoglutarate analogue, led to a similar change of tumour vessel structure or not. Changes of tumour vessel structure were also showed the same tendency with DMOG treatment, increased CD31-positive area, markedly increased average vessel diameter and decreased blood vessel density (Supplemental Fig. 1A–D).

### PHD inhibitor induced tumour vessel normalisation

**Figure 2:**
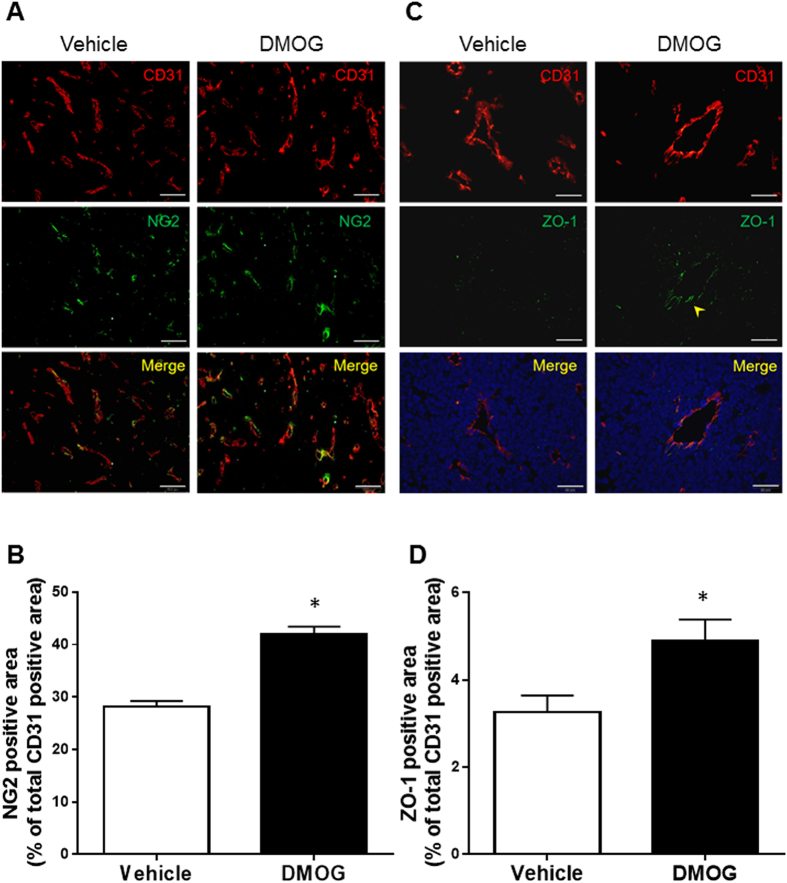
PHD inhibitors promote normal angiogenesis. The mice were injected subcutaneously with Lewis lung carcinoma tumour cells. At 10 days after the tumour cell injection, the mice were intraperitoneally administered dimethyloxalyl glycine (DMOG). Seven days after the DMOG administration, the mice were sacrificed, and the tumours were excised. (**A**) Representative immunofluorescence image of NG2 (green) and CD31 (red) co-staining. Scale bars represent 100 μm. (**B**) Quantification of the NG-2-positive area percentage in the CD31-positive area. (**C**) Representative immunofluorescence image of zonula occludens-1 (ZO-1) (green) and CD31 (red) co-staining. The arrow head indicates ZO-1-positive vessel. Scale bars represent 50 μm. (**D**) Quantification of the ZO-1-positive area percentage in the CD31-positive area. The bar charts show means ± s.e.m. These pictures were taken over 15 parts in each tumour. n = 3 mice per group. *Shows *p*-values that were <0.001 in (**B)** and <0.001 in (**D)**, respectively v.s. vehicle, Mann-Whitney U test.

We then evaluated whether or not those reconstituted tumour blood vessels had normal structures. In general, normal microvessels consisted of an EC layer covered with pericytes, and the ECs formed tight junctions between each other; however, tumour blood vessels were poorly covered with pericytes. Representative fluorescent images for NG-2, a marker for pericytes, are shown in Fig. 2A. In the quantification of the immunofluorescent image, DMOG-treated tumour vessels had a higher percentage of pericyte coverage (Fig. 2B). Moreover, the tight junctions in normal microvessels performed as an EC barrier, which inhibited the extravasation of large molecules (ex. M.W. >70 K). Tumour vessels had poor tight junctions, which were associated with tumour hypoxia and anti-cancer drug resistance. The vehicle control mice lacked zonula occludens-1 (ZO-1) which formed tight junctions of the ECs. The DMOG-treated mice not only had increased ZO-1 expression but also improved tight junction formation in the vessels (Fig. 2C,D). We also evaluated pericyte coverage and tight junction formation in FG4592-treated mice, which revealed markedly increased percentage of NG-2 coverage and tight junction formation (Supplemental Fig. 2A–D). These data suggest that PHD inhibitor treatment induces not only tumour vessel reconstitution but also improves tumour vessel maturation. Thus, PHD inhibitor treatment promotes vessel normalisation.

### Tumour vessel normalisation by PHD inhibitor repaired its perfusion and permeability

**Figure 3:**
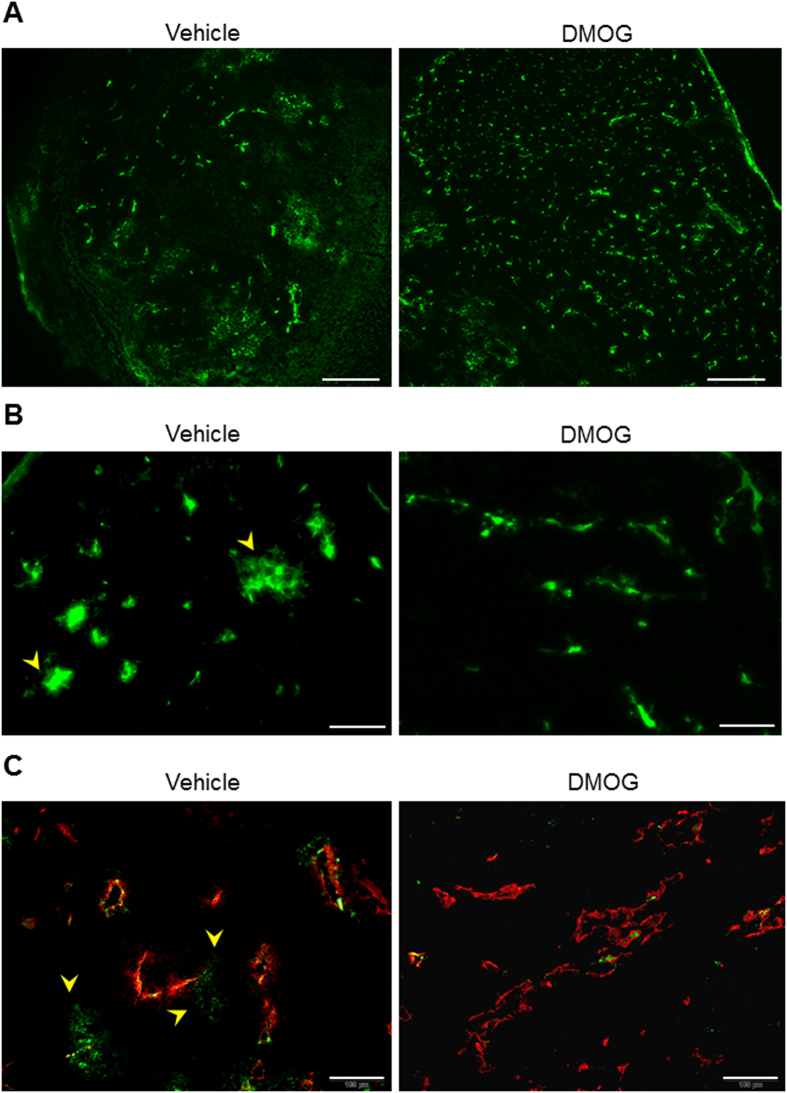
Improvement of vessel leakage after dimethyloxalyl glycine treatment. Seven days after treatment with or without dimethyloxalyl glycine (DMOG), Dextran–fluorescein isothiocyanate (FITC) was injected into the mice through the tail vein before sacrifice. (**A**) Representative fluorescence images of the tumour tissue perfusion with Dextran–FITC. Scale bars represent 500 μm. (**B**) Representative fluorescent images of the blood leakage in the tumour tissues. Scale bars show 100 μm. (**C**) Blood leakage images of co-staining with CD31 (red) in Dextran–FITC administered samples.

**Figure 4:**
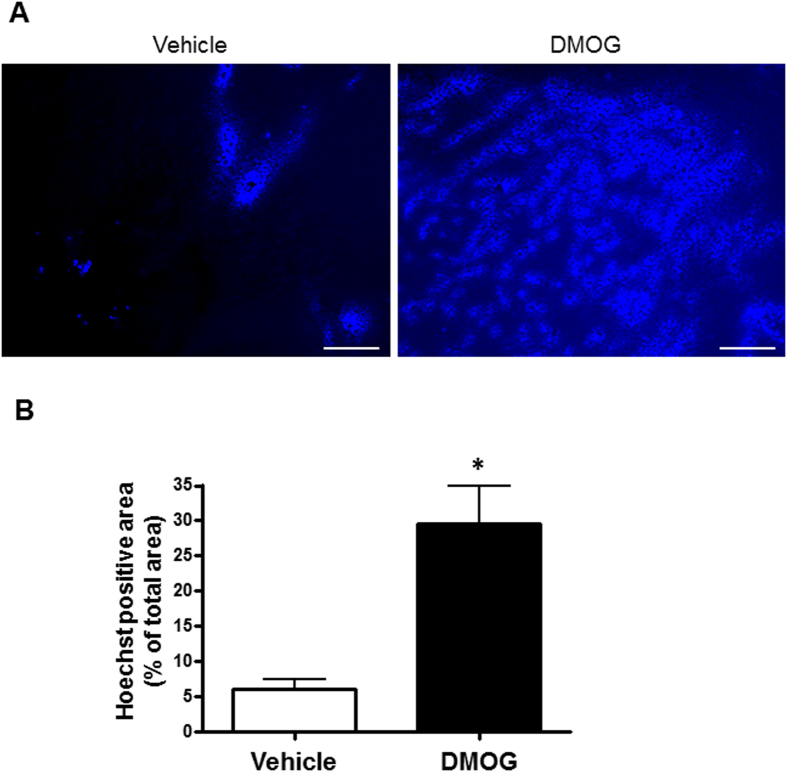
Improvement of drug distribution after dimethyloxalyl glycine treatment. (**A**) Representative images of drug distribution using Hoechst 33342. (**B**) Quantification of the Hoechst distribution area percentage. Bar charts represents means ± s.e.m. These pictures were taken over 15 parts in each tumour. n = 3 mice per group. *p-value was <0.001 v.s. Mann–Whitney U test.

To confirm whether the reconstituted tumour vessel was functionally normal, we evaluated the tissue blood perfusion, vessel leakage and drug distribution. We used high molecular weight fluorescence-labelled Dextran (200 K), which did not leak from normal vessels. Tumour tissue perfusion was recovered after DMOG treatment (Fig. 3A). The leakage of Dextran conjugated with fluorescein isothiocyanate (FITC; Dextran–FITC) was observed in the vehicle controls as a hazy green area (yellow arrow head); however, DMOG-treated tumour vessels were morphologically normalised and had recovered normal blood vessel functions, which prevented high weight molecules from penetrating the blood vessel (Fig. 3B,C). We also evaluated whether normalised blood vessels treated with DMOG showed improved drug delivery efficiency to tumour tissues. Hoechst33342 stain (M.W. = 562), which stains both live and dead cells, was substituted for an anti-cancer drug and stained tumour tissues to reflect perfusion and drug distribution. DMOG-treated tumours displayed dramatically improved drug distribution from the normalised tumour vessels compared with those from vehicle controls (Fig. 4A,B).

### Tumour hypoxia was improved by treatment with PHD inhibitor

**Figure 5:**
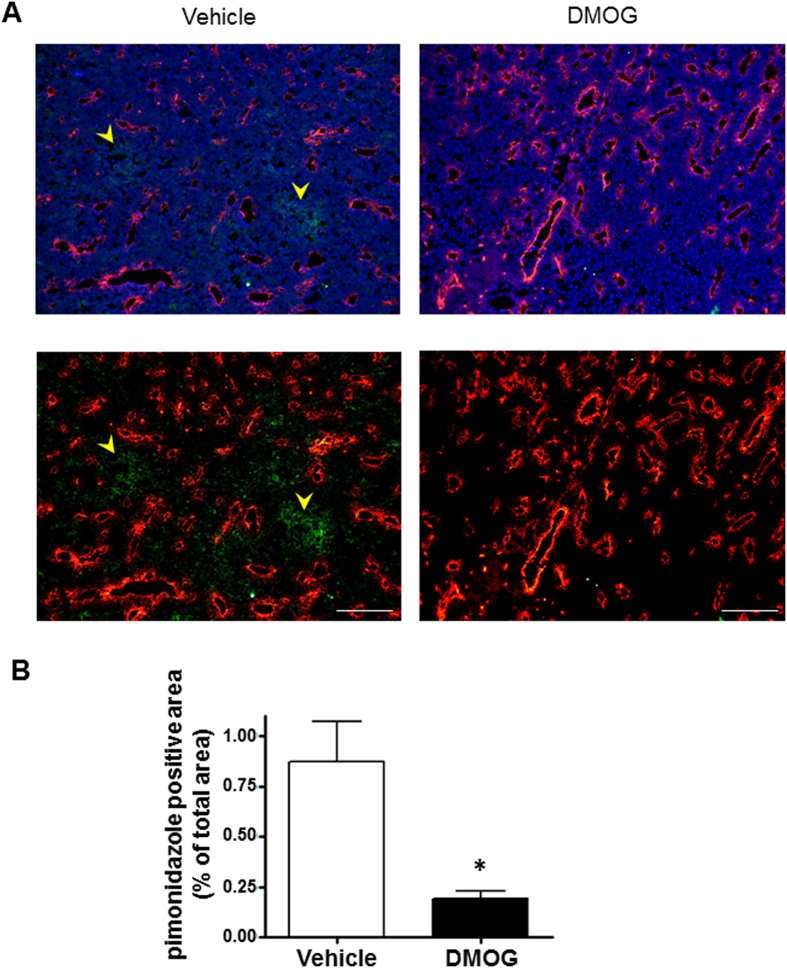
Tumour blood vessel normalisation reduces tissue hypoxia region. The mice were injected with the hypoxia probe. Seven days after treatment with or without dimethyloxalyl glycine (DMOG), mice were injected with 60 mg/kg pimonidazole to label the hypoxic regions. (**A**) Representative images of tumour hypoxic regions with immunofluorescence staining. Hypoxic regions and endothelial cells were stained with anti-pimonidazole antibody (green) and anti-CD31 antibody (red) and counterstained with DAPI (blue). (**B**) Quantifications of the pimonidazole-positive area ratio. Bar charts show means ± s.e.m. These pictures were taken over 15 parts of each tumour. n = 3 mice per group. *p-value was < 0.05 v.s. vehicle, Mann–Whitney U test.

Hypoxic regions, which were found in tumour tissues, were feasible environments for tumour progression, and tumour cells with chemo- and radio-resistance had a poor prognosis. To evaluate if vessel normalisation improved the hypoxic environment in the tumour, a hypoxia probe was administered to tumour-bearing mice to detect regions of tissue hypoxia. Hypoxic regions in the tumour tissue are shown with immunofluorescence staining in Fig. 5A. The pimonidazole-positive regions were dramatically reduced in the DMOG-treated mice. Morphological and functional vessel normalisation after DMOG treatment led to an improvement in oxygen distribution and a statistically significant reduction in tumour hypoxic regions (Fig. 5B).

### Reconstituted tumour tissues were sensitised to anti-cancer drugs

**Figure 6:**
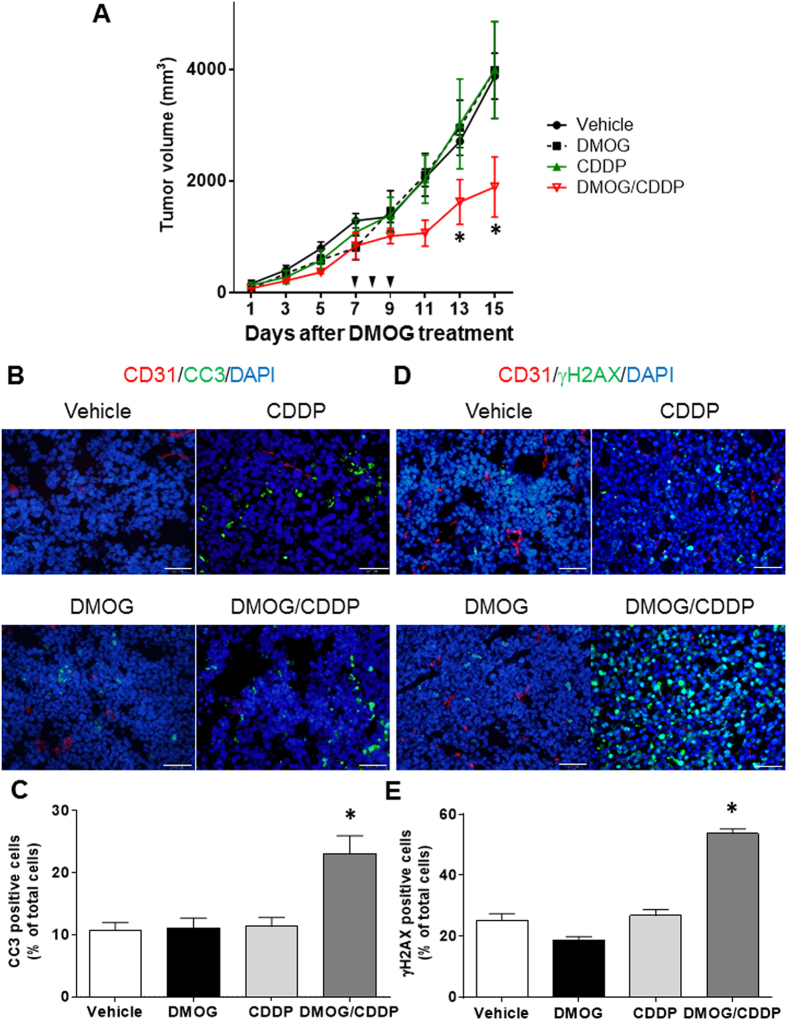
Dimethyloxalyl glycine treatment improved chemo-sensitivity. Seven days after treatment with or without dimethyloxalyl glycine (DMOG), each group was treated with or without cisplatin (CDDP) 2.5 mg/kg/day i.p. for 3 days. (**A**) The tumour growth curve for DMOG and CDDP with or without treatment. (**B**) Representative image of cleaved caspase-3 (green) and CD31 (red) immunofluorescence staining at 48 h after the final CDDP administration. Counterstained nuclei with 4′,6-diamidino-2-phenylindole (DAPI; blue). (**C**) Quantification of the cleaved caspase-3-positive cell ratio. Bar charts show means ± s.e.m. Over 10,000 nuclei were counted in each tumour tissue. n = 3 mice per group. *p < 0.05 v.s. vehicle, *t*-test. (**D**) Representative image of γH2AX (green) and CD31 (red) immunofluorescence staining at 48 h after the third CDDP administration. The nuclei were stained with DAPI (blue). (**E**) Quantification of the cleaved caspase-3 (CC3)-positive cell ratio. Bar charts show means ± s.e.m. Over 10,000 nuclei were counted in each tumour tissue. n = 3 mice per group. *p < 0.05 v.s. Vehicle, *t*-test.

To assess the anti-cancer drug activity, we evaluated the results of vessel normalisation and functional improvement after DMOG treatment. Typically, overexpression of HIFs is considered a bad prognostic factor; therefore, we assessed if DMOG treatment itself affects tumour growth, metastases, cell proliferation and survival term; consequently, DMOG treatment was not found to affect any of them (Supplemental Fig. 3A–D). The mice were randomly divided into four groups: vehicle, DMOG treatment, cisplatin (CDDP) treatment and DMOG/CDDP treatment. CDDP was administered for 3 days at 6 days after the DMOG injection. Treatment with DMOG alone did not affect tumour growth compared with vehicle. Treatment with CDDP alone did not reduce or inhibit tumour progression; however, the combination of DMOG and CDDP treatment temporally reduced or inhibited tumour progression (Fig. 6A). To assess the different therapeutic efficacies of these two groups, we performed an apoptosis and DNA damage analysis in each group. Apoptosis in tumour tissue was assessed with cleaved caspase-3 (CC3), and the DNA damage in cells was detected with γH2AX staining. The DNA damage and apoptosis significantly increased staining in both DMOG- and CDDP-treated mice; however, no significant difference was observed in any other group (Fig. 6B–E). These results showed that vessel normalisation induced an increase in chemo-drug sensitivity due to an improvement in drug distribution.

## Discussion

Our results demonstrated that tumour blood vessel normalisation accompanied by angiogenesis after treatment with PHD inhibitor led to the repair of sensitivity to chemotherapy based on the structural alterations in the tumour vessels and promoted tight junction formation and pericyte coverage.

Blood vessel ECs are equipped with oxygen sensors. The signalling pathways for HIFs, such as PHD2 and HIF2α, allow the vessels to readjust their shape to optimise blood flow^14^. Transient PHD regulation by DMOG and FG4592 activated the HIF pathways, which promoted angiogenesis and improved blood circulation in ischaemic tissue; HIF2α overexpression in ECs promoted vascularization and enhanced wound healing in skin in our previous study^13^,^15^. HIF2α in ECs has an important role in tumour angiogenesis, vessel lumen development and vessel sprouting^16^. In our study, PHD inhibitor treatment promoted angiogenesis and development of vessel lumens and decreased the number of vessels in the tumour. Furthermore, the tumour vessels had a heterogeneous distribution^17^, which also may be associated with decreased vessel density. PHD inhibitor induced angiogenesis and vasculogenesis, which led to remodelling of the tumour vasculature^10^; these may influence the increased vessel areas and dilated vessel diameters.

Lacks of pericytes and tight junction formation in the blood vessels are popular features of tumour vessels, unlike normal vessels. These were regulated by several factors, such as VEGF, which was controlled by PHD2 in the ECs^14^. We demonstrated that transient PHD inhibition by DMOG and FG4592 promoted pericyte coverage or migration to the vessels, tight junction formation, vessel maturation and barrier tightening. However, this treatment affected not only the ECs but also other cells in the entire tissue. Hence, it should be considered that various cells and factors influence ECs in tumour tissue. we could not identified vessel normalisation orchestrating cell and these detail molecular mechanism in this study; there could be a common mechanism with the wound healing process, the possibility of which should be considered in the next study.

These structural changes have often been accompanied by the normalisation of vessel function^18^, which formed the tight junctions of ECs and were covered by pericytes with smooth alignment. Tight junctions regulated the movement of solutes, ions and water across the paracellular space^4^. Therefore, a tight junction deficiency can lead to hyperpermeability of the ECs and can decrease nutrition or oxygen delivery in tissues^5^. These conditions can reduce blood circulation and oxygenation in the tumour tissue, accelerating the vicious circle in the tumour microenvironment. Tumour cells produce abnormal angiogenic factors, and normal ECs produce normal factors; therefore, the amount of each factor influences the level of productivity of tumour angiogenesis^1^,^18^. We demonstrated that transient inhibition of PHD decreases nonproductive angiogenesis and improves the tumour microenvironment. Overexpressed HIFs may promote both angiogenesis and vessel remodelling; however, the relative balance of these two factors likely even leans towards more productive angiogenesis. Therefore, this vessel normalisation led to reduced permeability, increased tissue perfusion and presumably increased blood flow in the tumour. Both these vessel normalisation phenomena induced oxygenation in tissue and improved hypoxia in tumour. Similar effects after vessel normalisation were also reported for anti-VEGF therapy in mice tumour models^19^,^20^; however, anti-VEGF therapies blocked neovascularisation and removed microvascular. From this perspective, anti-VEGF therapies are less useful in hypovascular tumours, such as pancreatic carcinoma. In contrast, a PHD inhibitor may normalise the vessels in any type of tumour with normal angiogenesis; consequently, the vessel normalisation induced by a PHD inhibitor could be more useful than that induced by anti-VEGF agents.

Tumour microenvironments enhance resistance to anti-cancer therapies and radiation therapy; many chemotherapeutics rely on oxygen radical formation to kill cancer cells, and tumour hypoxia reduces their efficacy^21^. Therefore, we believe that our strategy may be more convenient for clinical therapy of cancer. In our results, the vehicle, CDDP and DMOG treatment groups did not exhibit any inhibitory effect on tumour growth. However, the DMOG/CDDP treatment group showed significant inhibitory effects on the tumours. Furthermore, in this group, the tumour vessels were normalised by the time of CDDP administration; this normalisation may lead to improved chemotherapy efficiency. DMOG administration, which improved tissue perfusion, also improved tumour hypoxia by decreasing chemo-resistance without accelerating primary tumour growth. Consequently, it resulted in increased DNA damage and intra-tumour apoptosis accompanied by a reduction in tumour growth compared with any other group. This similar effect has been reported for tumour vessel normalisation after treatment with chloroquine^22^; however, this report revealed an augmentation in anticancer effects for small volume tumours of <1,000 mm^3^. In our study, CDDP administration was started when the mean tumour volume reached >1,000 mm^3^. In general, larger tumours exhibit more resistance to anticancer therapies than smaller tumours^23^,^24^. Thus, PHD inhibitor administration and vessel normalisation could be more effective in large, advanced cancers. In addition, the dosage of CDDP could be reduced from the present clinical dose; therefore, it might contribute to the reduction of adverse events associated with cancer chemotherapy.

Originally, we expected that PHD inhibitor treatment would promote tumour growth, but surprisingly, the growth was not stimulated after PHD treatment. The PHD inhibitory effect appeared to be transient; however, normalised vessels induced the oxygenation of the ischaemic tumour tissue, which enhanced the activation of innate and adaptive immune responses and cell death programs^25^. Therefore, ischaemia reperfusion may be one of the reasons that the tumour growth rate of the DMOG-treated group was the same as that of the vehicle group. Moreover, another study of tumour vessel normalisation through PHD2 inhibition showed results that were similar to those of our study; this relied on the antiproliferative nature of the TGFβ-signalling pathway in a largely HIF-independent manner^26^.

In this study, we found that tumour vessel normalisation after the administration of a PHD inhibitor enhanced the efficacy of chemotherapy without accelerating the primary tumour growth. It may be an effective new strategy for anticancer treatments. In addition, it may be more beneficial for the treatment of large advanced cancers without increasing chemotherapy-associated adverse events.

## Materials and Methods

### Animals

All animal experiments were approved by the Institutional Animal Care and Use Committee of Tottori University (approved number: 14-Y-12) that was approved by the Japanese Association for Accreditation for Laboratory Animal Care, and the animal research and handling were performed in strict conformance with federal Institutional Animal Care and Use Committee guidelines.

The C57BL/6 female mice were obtained from CLEA Japan, Inc. and housed in cages with food and water available *ad libitum* in a 12-h light–dark cycle at 22 ± 1 °C.

### Cell cultures, tumour transplant model and PHD inhibitor treatment

The LLC cells were maintained in Dulbecco’s Modified Eagle Medium containing 10% foetal bovine serum and penicillin/streptomycin in 5% CO_2_ and 95% room air at 37 °C.

These cells were harvested and re-suspended (at 1 × 10^7^ cells/mL) in phosphate-buffered saline (PBS). Some of the cells (1 × 10^6^ cells) were subcutaneously transplanted into the right flank of the mice which were aged at 8–12 weeks. The mice were treated with 400 mg/kg DMOG (Cayman Chemical, MI, USA) or 50 mg/kg FG4592 (Cayman Chemical, MI, USA) intraperitoneally 10 days after the tumour transplant. Once every two days, the tumours were measured in two dimensions using a calliper. The tumour tissue volume was calculated using the formula: V = π (length × width^2^)/6. The mice were sacrificed at a defined time point or when the tumour volume reached 4500 mm^3^.

### Immunofluorescence staining

The tumour tissues were sliced into frozen sections of 8-μm thickness at −20 °C and were dried in air. The sectioned tissue samples were rehydrated with PBS for 10 min and fixed with 4% (w/v) cold paraformaldehyde for 10 min. The sections were washed with PBS and permeabilised with 0.5% (v/v) Triton^TM^ X-100 in PBS for 10 min. The sections were blocked in 5% normal goat serum for 30 min at room temperature. The sections were incubated with the following primary antibodies: anti-CD31 (1:500) (eBioscience, CA, USA), anti-NG2 (1:400) (Merck Millipore, MA, USA), anti-ZO-1 (1:400) (Thermo Fisher Scientific, MA, USA), anti-CC3 (1:400) (Cell Signalling Technologies, MA, USA) and anti-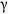
H2AX (1:1000) (Merck Millipore, MA, USA) overnight at 4 °C. The sections were washed with 0.1% Tween 20 in PBS and incubated for 1 h at room temperature with the appropriate fluorophore secondary antibody (AlexaFluor 488 or Cy3, goat anti-rat, or goat anti-rabbit IgG; Biolegend, CA, USA). The sections were washed with 0.1% Tween 20 in PBS, dehydrated with ethanol and dried in air; they were then mounted with the Vectashield mounting medium (Vector Laboratories, CA, USA) containing 4′,6-diamidino-2-phenylindole stain (1:5000) and a cover slip.

### Quantification of immunofluorescence images

The quantification of the immunofluorescent images which were taken using a microscope (BZ-9000, KEYENCE, Osaka, Japan) was performed using its application software. At least 15 fields of images at 100, 200 or 400 magnification were analysed for each sample. Each experiment used at least three animals from each group.

### Tumour vessel perfusion and permeability analysis

Dextran–FITC (Sigma-Aldrich, MO, USA) was intravenously administered to a tail vein 10 min prior to sacrifice. The tumour tissues were excised and immediately frozen with liquid nitrogen. The tumour samples were stored at −80 °C until the sectioning. The tumour tissues were partitioned into sections of 8- and 20-μm thickness and were observed using a fluorescent microscope. Furthermore, the 8-μm sections were prepared for immunofluorescence staining with anti-CD31 antibody and were evaluated for vessel permeabilisation.

### Drug distribution analysis

Drug distribution in the tumour tissues was evaluated using Hoechst 33342 (Dojindo laboratories, Kumamoto, Japan). The tumour-bearing mice, which had been treated with or without DMOG 6 days prior, were injected with Hoechst 33342 50 mg/kg in PBS into the tail vein 10 min prior to sacrifice. The tumours were excised and immediately frozen with liquid nitrogen. The tumour samples were stored at −80 °C until sectioning. The tumour tissues were then partitioned into frozen sections of 20-μm thickness. The sectioned samples were observed under a fluorescent microscope, images were obtained and we quantified the percentage of the Hoechst-positive area.

### Detection of tumour hypoxia region and analysis

The hypoxic regions of the tumour tissues were detected using the Hypoxyprobe™ kit (Hypoxyprobe Inc. MA, USA), which included pimonidazole, as per the manufacturer’s protocol. In brief, pimonidazole was injected at 60 mg/kg into the tumour-bearing mice. Ninety minutes later, the mice were sacrificed; we collected the tumours and cryosectioned the tissue into 4-μm thick slices. The sections were fixed with ice-cold acetone for 10 min, washed with PBS and then incubated with rabbit anti-pimonidazole anti-sera (1:20) overnight at 4 °C. The sections were then incubated with AlexaFluor 488 conjugated goat anti-rabbit antibody (1:1000) for 1 h. The images were obtained with the KEYENCE BZ-9000 microscope, the ratio of the pimonidazole-positive area was quantified using the equipped software.

### Anticancer drug treatment

The sensitivity of cancer chemotherapy was assesses with CDDP administration. Tumour-bearing mice had been treated with or without DMOG; 6 days later, these mice were treated with or without 2.5 mg/kg cisplatin (Yakult, Tokyo, Japan) intraperitoneally for 3 days in a row. The tumours were measured once every two days after the DMOG administration until a defined time point. The tumour volume was calculated using the formula described above. The apoptosis and DNA damage analysis in the tumour tissue was evaluated with CC3 and γH2AX at 48 h after the final administration of CDDP. The nuclei of the tumour cells that had been treated with CC3, and the γH2AX foci were quantified as a percentage of the total nuclei within 15 fields of view in a magnification of 400.

### Statistical analysis

Statistical analyses were performed using the unpaired t-test followed by a Bonferroni’s post-hoc test. All statistical analyses were performed using the GraphPad Prism (version 6.02) software. Statistical significance was defined as a *p* value of <0.05, but values of *p* < 0.01, *p* < 0.001 and *p* < 0.0001 are also shown to indicate the level of confidence.

## Additional Information

**How to cite this article**: Koyama, S. *et al*. Tumour blood vessel normalisation by prolyl hydroxylase inhibitor repaired sensitivity to chemotherapy in a tumour mouse model. *Sci. Rep.* **7**, 45621; doi: 10.1038/srep45621 (2017).

**Publisher's note:** Springer Nature remains neutral with regard to jurisdictional claims in published maps and institutional affiliations.

## Supplementary Material

Supplemental Information
