# Supplementary material for: Tumour blood vessel normalisation by prolyl hydroxylase inhibitor repaired sensitivity to chemotherapy in a tumour mouse model
Source: Sci Rep. 2017 Mar 31;7:45621. doi: 10.1038/srep45621 (PMC5374523; doi:10.1038/srep45621)
Supplement: Supplemental Information [file srep45621-s1.pdf]

Supplemental information

Tumour blood vessel normalisation by prolyl hydroxylase inhibitor repaired sensitivity to chemotherapy in a tumour mouse model

Satoshi Koyama[1,2], Shinji Matsunaga[1,4], Masaki Imanishi[1], Yoichi Maekawa[3], Hiroya Kitano[2], Hiromi Takeuchi[2], and Shuhei Tomita[1,4,*]

[1]Division of Molecular Pharmacology, Department of Pathophysiological and Therapeutic Science, Tottori University Faculty of Medicine, Japan

[2]Division of Otolaryngology, Head and Neck Surgery, Department of Sensory and Motor Organs, Tottori University Faculty of Medicine, Japan

[3]Department of Parasitology Gifu University Graduate School of Medicine, Japan

[4]Department of Pharmacology, Osaka City University Graduate School of Medicine, Japan

*E-mail: tomita.shuhei@med.osaka-cu.ac.jp

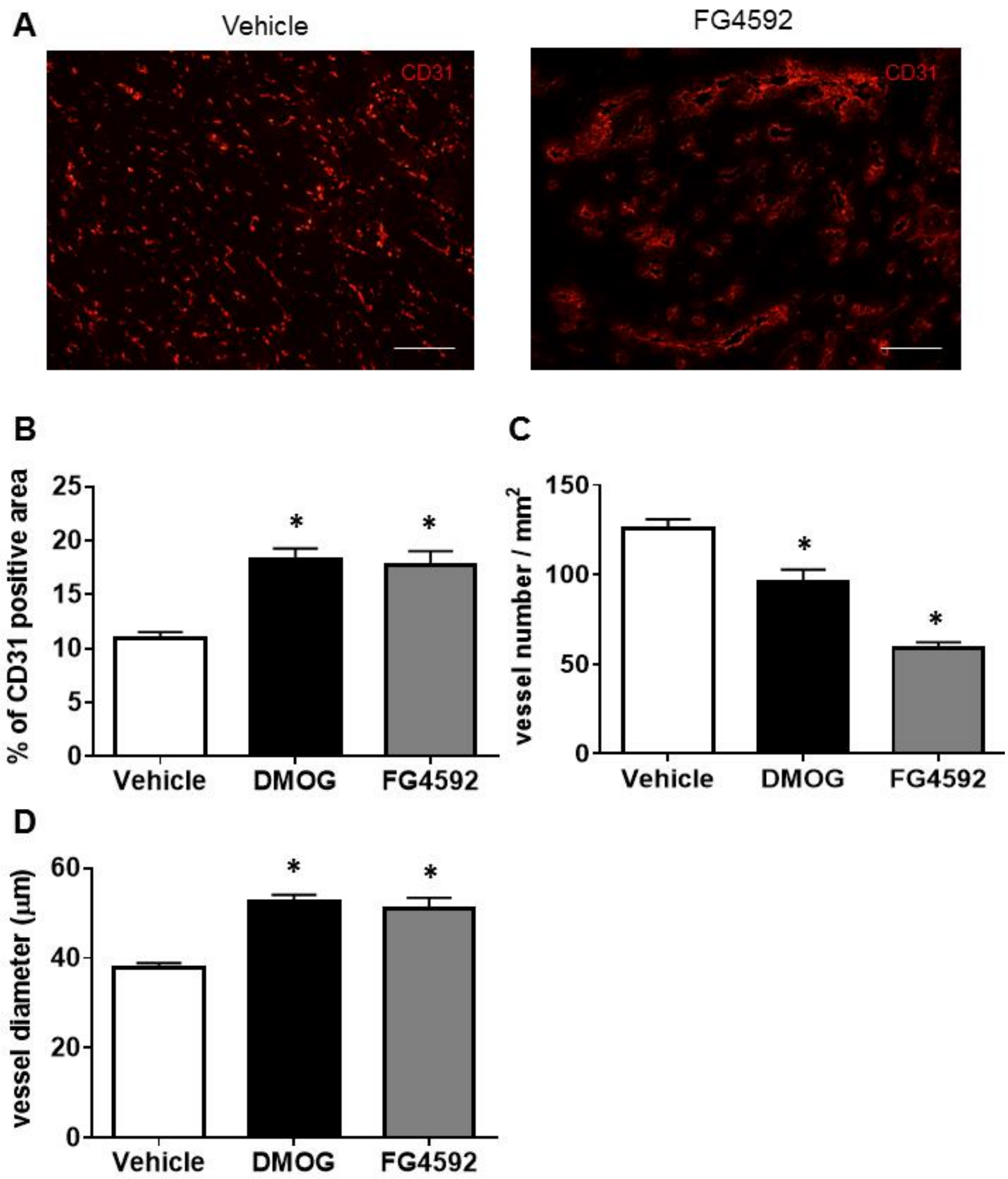

Supplementary Figure S1. Another PHD inhibitor FG4592 changed the tumour vessel structure in the same way as DMOG administration

A) Another PHD inhibitor FG4592 was administrated with 50 mg/kg intraperitoneally 10 days after the tumour transplant. FG4592 treatment changed tumour vessel structure in the same as DMOG administration, which decreased vessel number and dilated vessel diameter.

B) CD31-positive area was significantly increased with administration of both PHD inhibitors—DMOG and FG4592.

C) Tumour vessel number was significantly decreased with administration of both PHD inhibitors—DMOG and FG4592.

D) Tumour vessel diameter was significantly increased with administration of both PHD inhibitors—DMOG and FG4592.

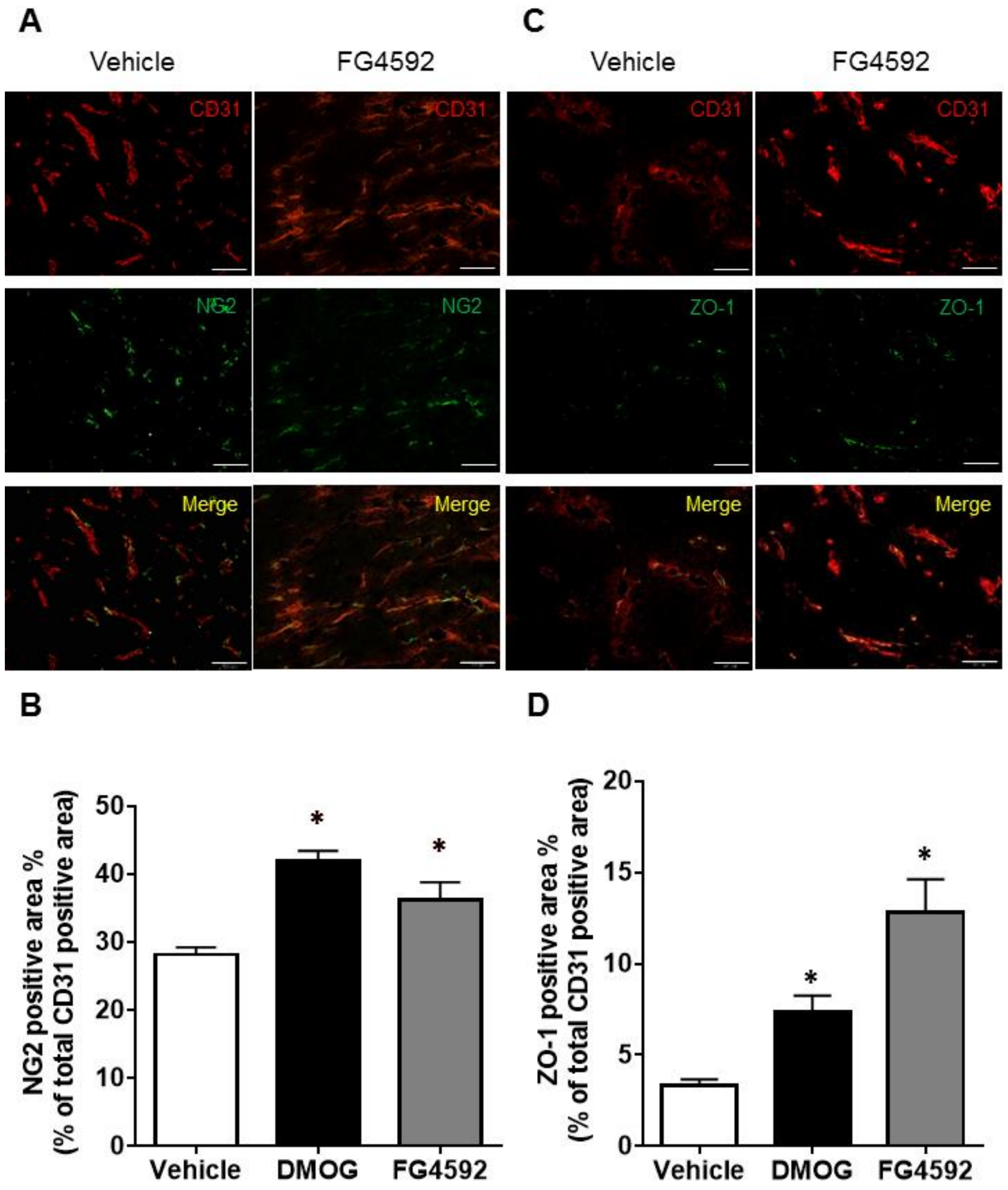

Supplementary Figure S2. Another PHD inhibitor FG4592 induced tumour vessel normalisation

A) Representative fluorescent images for CD-31 and NG-2 reveal increased pericyte coverage in FG4592-treated mice.

B) The quantification of NG-2 reveals higher percentage of pericyte coverage in FG4592-treated mice.

C) Representative fluorescent images for ZO-1 and CD31 reveal increased tight junction formation in FG4592-treated mice

D) The quantification of ZO-1 reveals higher percentage of tight junction formation in FG4592-treated mice

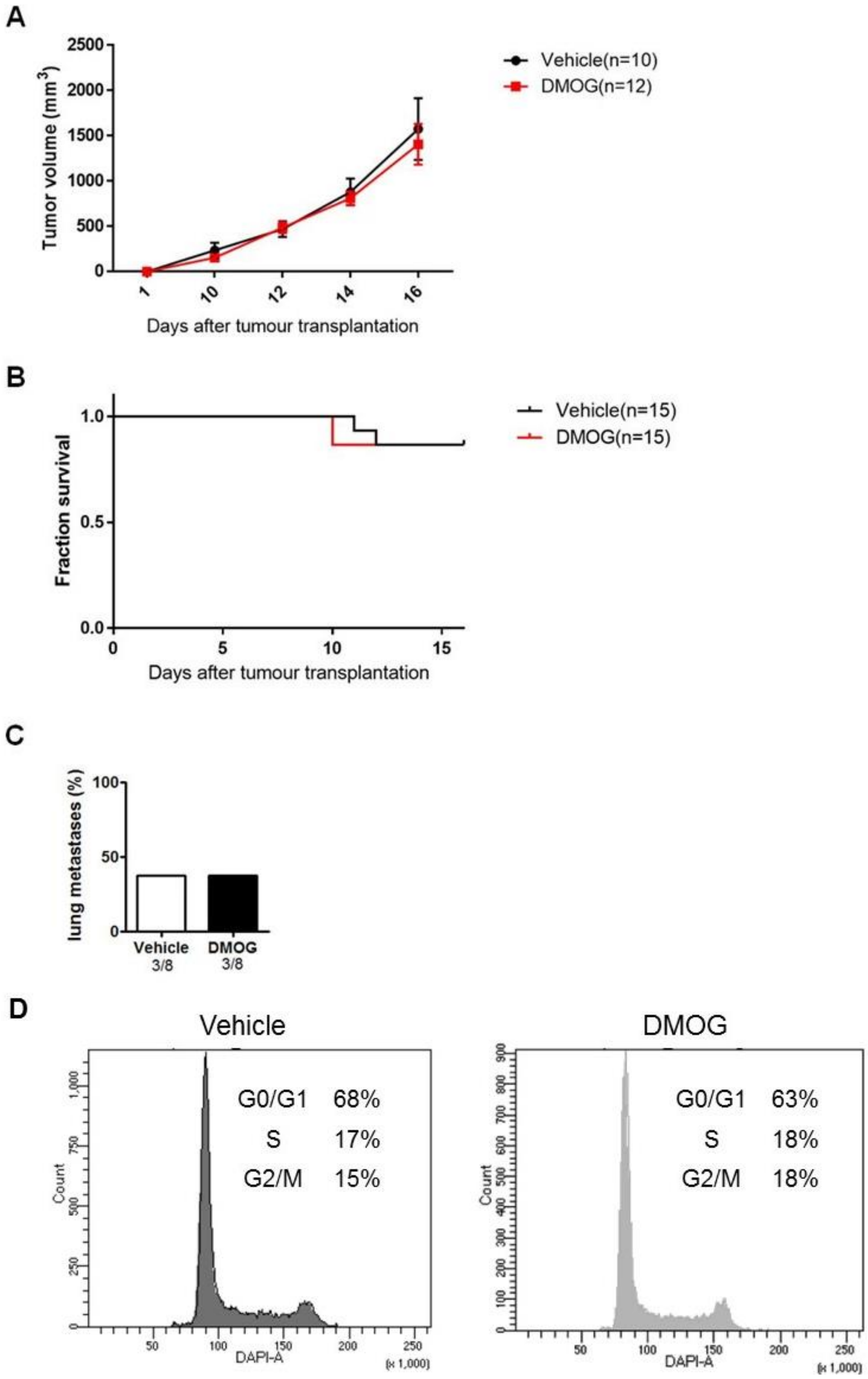

Supplementary Figure S3. Tumour growth, survival, and lung metastases were not affected by DMOG administration

A) DMOG administration does not affect the tumour growth rate (N = 10).

B) Kaplan–Meier survival curve illustrates that DMOG does not affect the survival ratio (N = 10).

C) Positive or negative lung metastases are macroscopically counted in HE-stained lung tissue (N = 8). DMOG does not affect the number of metastases.

D) Cell cycle analysis by using flow cytometry revealed that DMOG administration did not affect cell cycle population.
